# Epidemiology of traffic injuries in Iran-2017 

**Published:** 2019-08

**Authors:** Fatemeh Sadat Asgarian, Hossein Akbari, Mehrdad Mahdian

**Affiliations:** ^*a*^Trauma Research center, Kashan University of Medical Sciences, Kashan, Iran.; ^*b*^Social Determinants of Health (SDH) Research Center, Kashan University of Medical Sciences, Kashan, Iran.

## Abstract

**Background:**

Policies and strategies to improve health should be specified. For such policies and strategies, accurate information and indicators should be considered. To increase life expectancy and reduce mortality at different ages, causes of destroying life should be recognized and to be dealt with. Study design: In this study, accidents happened in Iran were investigated in year2017 to provide better directions to authorities in order to conduct appropriate planning and prevent occurrence of the majority of accidents in the country.

**Methods:**

This study was carried out based on secondary data analysis. Data gathered from outpatients or inpatients injured in accidents and referred to hospitals affiliated to all Universities of Medical Sciences registered in Health ministry of Iran was used. Data were analyzed using SPSS version 20.0 (SPSS Inc, Chicago, IL, USA), and statistical analyses were carried out with descriptive statistics, frequency, mean, and standard deviation. The Chi square test and independent t-test were used to determine association between variables.

**Results:**

In this study, 1,482,435 incidents were recorded. The majority of injured people were males. The highest percentage of accidents is attributed to traffic trauma. The most cases of injuries were related to urban areas. The incident was 18.5 per thousand and the highest incidence was related to Qazvin province with about 46.3 per thousand and then Kermanshah province with 38.1 per thousand. Tehran province alone accounted for 19.1% of all injured. The highest and lowest frequency of the incident occurred in the months of Mordad (August) (10.4%) and Bahman (February) (6.3%).

**Conclusions:**

Since half of the accidents occurred in younger age groups, it is necessary to provide accurate training to younger people. According to the results of this study, more accidents were happened in summer and outskirts, and it is suggested that these variables be considered more in future plans to reduce mortality from road accidents.

**Keywords:**

Iran, Epidemiology, Injury

